# Comparative genomics of carbapenem-resistant *Acinetobacter baumannii* isolated from pediatric patients in a tertiary care hospital

**DOI:** 10.1128/spectrum.01676-25

**Published:** 2025-10-06

**Authors:** Dafne Guillén-Navarro, Sara A. Ochoa, Daniela De La Rosa-Zamboni, Silvia Giono-Cerezo, Juan Xicohtencatl-Cortes, Ariadnna Cruz-Córdova

**Affiliations:** 1 Laboratorio de Investigación en Bacteriología Intestinal, Hospital Infantil de México Federico Gómez 61670, Ciudad de Mexico, Mexico; 2 Departamento de Microbiología, Instituto Politécnico Nacional, Escuela Nacional de Ciencias Biológicas 603017 , Ciudad de Mexico, Mexico; 3 Posgrado en Ciencias Quimicobiológicas, Escuela Nacional de Ciencias Biológicas, Instituto Politécnico Nacional 61735 , Ciudad de Mexico, Mexico; 4 Laboratorio de Investigación en Inmunoquímica, Hospital Infantil de México Federico Gómez 61670, Ciudad de Mexico, Mexico; 5 Departamento de Epidemiología, Hospital General de México Dr Eduardo Liceaga 61575 , Ciudad de Mexico, Mexico; Icahn School of Medicine at Mount Sinai, New York, New York, USA

**Keywords:** genomic, *Acinetobacter baumannii*, carbapenem, resistant, tertiary care hospital

## Abstract

*Acinetobacter baumannii* is a short gram-negative bacillus, notable for its intrinsic multidrug resistance and genomic plasticity, which facilitates the acquisition of additional resistance genes via mobile genetic elements. Due to its increasing carbapenem resistance, the World Health Organization has classified it as a critical priority pathogen. This study performed a comparative genomic analysis of 20 carbapenem-resistant *A. baumannii* clinical strains isolated from the Hospital Infantil de México Federico Gómez (CRAB-HIMFG), alongside 11 genomes from other Mexican strains. The pangenome was determined to be open, and core genome single-nucleotide polymorphism-based analysis grouped the CRAB-HIMFG strains within CC758/IC5 and CC92/IC2. A novel sequence type (ST) in the MLST-Pasteur scheme was identified, related to ST^Pas^156, and in the MLST-Oxford scheme, associated with ST^Oxf^758 and ST^Oxf^1054. Virulence and resistance genes comprised 0.61% to 2.23% of the pangenome. Oxacillinase genes and efflux pumps primarily mediated carbapenem resistance, while virulence genes included those encoding biofilm and type IV pili. Capsule typing revealed a correlation with established international clones, IC2 and IC5. Plasmids exhibited high diversity, harboring maintenance modules and toxin-antitoxin systems, with the dissemination of resistance genes linked to insertion sequences. Biofilm formation and twitching motility were not always expressed, as they depend on additional environmental factors. Our study shows that comparative genomics is an essential tool to analyze clinically and epidemiologically significant genomes, providing critical insights into gene distribution, genomic architecture, and horizontal gene transfer mechanisms in microbial populations.

**IMPORTANCE:**

In recent years, a reported increase in the mortality rate associated with infections caused by *A. baumannii*, along with a rise in carbapenem resistance, poses a serious clinical challenge. The WHO considered this microorganism critical for research into alternative therapies and epidemiological surveillance. Despite advances in bioinformatics, genomic studies have yet to fully elucidate the structural rearrangements and secretion systems of *A. baumannii*. This knowledge gap hinders our understanding of its remarkable genomic plasticity and its ability to acquire and spread resistance and virulence genes through horizontal gene transfer.

## INTRODUCTION

*A. baumannii* is a gram-negative, aerobic, and non-spore-forming bacillus with oxidative metabolism. It is oxidase-negative and catalase-positive. This bacterium is considered multidrug-resistant (MDR) and is known to cause healthcare-associated infections (HCAIs) (1). In Mexico, according to the second-semester report from the Red Hospitalaria de Vigilancia Epidemiológica (RHoVE) in 2024, *A. baumannii* was the sixth most isolated microorganism (1,820/28318) and the seventh *A. calcoaceticus - A. baumannii complex* (1,455/28318) (2). The most common infections caused by *A. baumannii* include pneumonia associated with mechanical ventilation and the use of a central venous catheter. Less common infections caused by this bacterium include skin and soft tissue infections, surgical site infections, and infections associated with urethral catheters (3, 4). Additionally, *A. baumannii* has also been found to cause community-associated infections (CAIs) in patients with comorbid conditions, such as alcoholism, cancer, diabetes, and chronic obstructive pulmonary disease (1, 3).

In Mexico, the mortality rate from *A. baumannii* infections is about 14.5% (5). Most strains are known by their resistance to carbapenems, including meropenem and imipenem, and they contain genes that encode carbapenemases, such as OXA-23, OXA-239, and OXA-58 (5). β-lactamases, according to Ambler’s classification based on amino acid sequence, are divided into four molecular classes: A, B, C, and D. Over 400 types of class D β-lactamases, known as OXAs, have been identified (6). The most prevalent in *A. baumannii* are OXA-23, OXA-24, and OXA-58 (1, 7). The mechanism of action of carbapenems involves catalyzing the hydrolysis of the β-lactam substrate, leading to the formation of an intermediate covalent bond with the acyl-enzyme complex, which is linked to a serine residue at the active site of *A. baumannii*. Carbapenems are considered the treatment of choice for *A. baumannii* infections (6, 8); however, increasing resistance has led to exploration of alternative treatments, such as antibiotic combinations, bacteriophages, and combined treatments involving both (9).

A comparative genomics study involves searching for genes, classification, regulatory analysis, and investigation of gene loss, duplication, horizontal gene transfer (HGT), and pangenome dynamics (10, 11). For *A. baumannii*, the pangenome comprises approximately 20,000 genes, while the core genome consists of about 2,200 genes (12–14).

Accessory genome variation is a powerful tool, particularly for studying microevolutionary timescales. Combined analysis of core and accessory genomes enhances the resolution of transmission dynamics for MDR and extensively drug-resistant (XDR) strains (15). The first genomic characterization of the *bla*_OXA-143-like_ oxacillinase family found in carbapenem-resistant *A. baumannii* from Mexico belonged to the international clone (IC) 2 and the Latin America endemic IC5, with most exhibiting an XDR profile (16). Plasmids play a critical role in facilitating the horizontal transfer of resistance genes among *A. baumannii* clones, accelerating antimicrobial resistance and dissemination in hospitals. Notably, the *bla*_OXA-72_ gene, a key carbapenem resistance gene, has been detected in pandrug-resistant *A. baumannii* strains from different clones and Mexican regions (17). Recently, phylogenomic analyses have identified four major ICs of *A. baumannii* in Mexico (IC1, IC2, IC5, and IC7), each linked to specific genetic traits, including sequence types (STs), capsule loci (KL), outer core loci (OCL), and antibiotic resistance profiles. The IC2 and IC5 clones are particularly widespread across Mexico, carrying distinctive markers such as oxacillinases OXA-66 and OXA-65, respectively (18).

This study was conducted as a comparative genomic analysis of carbapenem-resistant *A. baumannii* (CRAB) strains isolated from pediatric patients at the Hospital Infantil de México Federico Gómez (HIMFG). The aim was to analyze the relationships between CRAB strains and others previously sequenced in Mexico, identifying resistance genes, virulence factors, and mobile genetic elements (MGEs), while investigating phenotypic traits through *in vitro* assays.

## RESULTS

### Carbapenem-resistant *A. baumannii* HIMFG strains were isolated from patients with acute lymphoblastic leukemia as the underlying disease

Twenty carbapenem-resistant *A. baumannii* HIMFG (CRAB-HIMFG) strains were isolated from pediatric patients between 2015 to 2017 from different samples. Among these, 35% (7/20) came from blood cultures; 15% (3/20) each from bronchial aspirates and catheter cultures; 10% (2/20) from stool cultures; and 5% (1/20) each from bone marrow aspirate, urine culture, cerebrospinal fluid, peritoneal dialysis fluid, and cholesteatoma cultures. The CRAB-HIMFG strains were isolated from patients hospitalized in the following wards: 55% (11/20) in the intensive care unit (ICU); 15% (3/20) in nephrology; 10% (2/20) each in cardiology, emergency room; and 5% (1/20) each in internal medicine and neurology (Table 1).

**TABLE 1:** Clinical characteristics of CRAB-HIMFG strains^*a*^

Patient	Age	Sex	Strain	Year	Source	Ward	Underlying disease
P1	2	Female	169D	2015	Bronchial aspiration	ICU	n/a
P2	12	Male	209D	2015	Peritoneal dialysis fluid	ICU	ALL2
			810CP		Stool culture		
P3	10	Male	471CP	2015	Stool culture	Cardiology	Non-Hodgkin’s lymphoma
P4	17	Male	769BC	2015	Blood culture	ICU	ALL2
			800BC				
P5	1	Male	139BC	2016	Blood culture	Emergency Room	n/a
P6	12	Female	361BC	2016	Blood culture	ICU	ALL2
P7	6 months	Male	728D	2016	Catheter culture	ICU	Nosocomial pneumonia and Gaucher disease
P8	1	Male	916D	2016	Catheter culture	Internal Medicine	Polytrauma
P9	6	Male	144D	2017	Bronchial aspiration	Nephrology	Nosocomial pneumonia
			658U		Urine culture		
P10	2	Male	241D	2017	Bronchial aspiration	Emergency Room	Epilepsy
P11	9	Male	580D	2017	Bone marrow aspirate	ICU	ALL2
			600BC		Blood culture		
P12	5 months	Female	721D	2017	Catheter culture	Cardiology	Interventricular communication with bidirectional short circuit
P13	2	Male	815D	2017	Cholesteatoma culture	Nephrology	Otomastoiditis with infiltration
P14	1	Male	940BC	2017	Blood culture	ICU	Neuroinfection
			966CSF		Cerebrospinal fluid		
P15	13	Male	986BC	2017	Blood culture	Neurology	Community-acquired pneumonia

^*a*^ BC, blood culture; D, diverse fluids; CP, stool culture; CSF, cerebrospinal fluid; ICU, intensive care unit; ALL2, acute lymphoblastic leukemia; n/a, not available data.

The strains were obtained from 15 patients, whose average ages were distributed as follows: 53.33% (8/15) were aged 0 to 4 years; 6.67% (1/15) were aged 5 to 8 years; 26.67% (4/15) were aged 9 to 12 years; and 13.33% (2/15) were aged 13 to 17 years. Among the CRAB-HIMFG strains, 80% (12/15) were isolated from male patients and 20% (3/15) from female patients.

The underlying diseases of these patients were distributed as follows: 26.67% (4/15) had acute lymphoblastic leukemia (ALL2); 13.33% (2/15) presented nosocomial pneumonia (one of them also had Gaucher disease); 6.67% (1/15) each had non-Hodgkin’s lymphoma, polytrauma, epilepsy, interventricular communication with a bidirectional shunt, otomastoiditis with infiltration, community-acquired pneumonia, and neuroinfection; and 13.33% (2/15) had not available data (Table 1). Regarding patient outcomes, 46.67% (7/15) died, 33.33% (5/15) were discharged, 6.67% (1/15) remained under clinical follow-up, and 13.33% (2/15) had no available outcome data.

### Genomic analysis

#### Genome assembly of CRAB-HIMFG

In this study, 20 CRAB-HIMFG strains collected between 2015 to 2017 from pediatric patients were included. Nineteen strains were sequenced using both the Illumina NextSeq 500 and MinION Oxford Nanopore platforms. The sequenced strains were registered at NCBI BioProject PRJNA1258274. The 810CP strain had been previously sequenced (19). Genome sizes of the strains ranged from 3.9 to 4.4 Mb, while plasmid sizes varied from 2.8 to 316.3 Kb (Table S1). The average nucleotide identity (ANI) values based on the BLAST algorithm (ANIb) and the MUMer algorithm (ANIm) were both >97%, and the tetra-nucleotide analysis (TETRA) score was >0.997. These results, which are consistent with previous reports (20), confirm that our strains belong to the same species, *A. baumannii*.

The comparative genomic analysis included 11 *A*. *baumannii* genomes from the BV-BRC database (AB-DB) (Bacterial and Viral Infectious Disease Research v.3.44.4 database https://www.bv-brc.org/; consulted on August 15, 2023) (21). The AB-DB genomes were obtained from human samples between 2006 and 2013 across seven Mexican states (Table 2), with the following sample distribution: 54.55% (6/11) from bronchial aspirates; 18.19% (2/11) from tissue cultures; and 9.10% (1/11) each from colon biopsies, wound cultures, and blood cultures. Regarding the age distribution of patients in the AB-DB, 9.10% (1/11) were aged 18 to 30 years; 18.19% (2/11) were aged 31 to 40 years, with additional cases in the 41 to 50 and 51 to 60 age groups. Age data were unavailable for 36.36% (4/11) of the strains. Sex distribution was as follows: 45.45% (5/11) of the patients were male, 36.36% (4/11) were female, and 18.19% (2/11) had no sex data available (Table 2). Genome sizes ranged from 3.9 to 4.3 Mb, and the plasmid sizes varied between 5.3 and 170.4 Kb (Table S1).

**TABLE 2:** Clinical information of AB-DB genomes from the BV-BRC database^*a*^

Strain	Strains information			Patient information	
	Year	Sample	State	Age	Sex
7804	2006	Bronchial aspiration	Nuevo León	25	Male
7835	2007	Bronchial aspiration	Nuevo León	n/a	Male
7847	2008	Tissue	Nuevo León	n/a	Female
3207	2008	Bronchial aspiration	Nuevo León	45	Female
5845	2009	Wound	San Luis Potosí	57	Male
AF-401	2009	Colon biopsy	n/a	n/a	n/a
9102	2010	Bronchial aspiration	Baja California	46	Female
10042	2011	Tissue	Coahuila	51	Female
11510	2012	Bronchial aspiration	Mexico City	n/a	n/a
10324	2012	Bronchial aspiration	Guerrero	31	Male
9201	2013	Blood Culture	Jalisco	37	Male

^*a*^ n/a, not available data.

#### Pangenome analysis revealed an open state in *A. baumannii*

The pangenome analysis of *A. baumannii* strains (CRAB-HIMFG and AB-DB) allowed us to estimate both genome sizes and gene repertoires. The pangenome was classified as open, meaning it can expand indefinitely with the addition of new genomes, enabling the acquisition of novel accessory genes (Fig. S1A). Conversely, the core genome decreases progressively with the addition of each genome (Fig. S1A). Using Roary software (22) for gene cluster identification, based on the presence or absence of genes, 10,033 gene clusters were identified.

**Fig 1:**
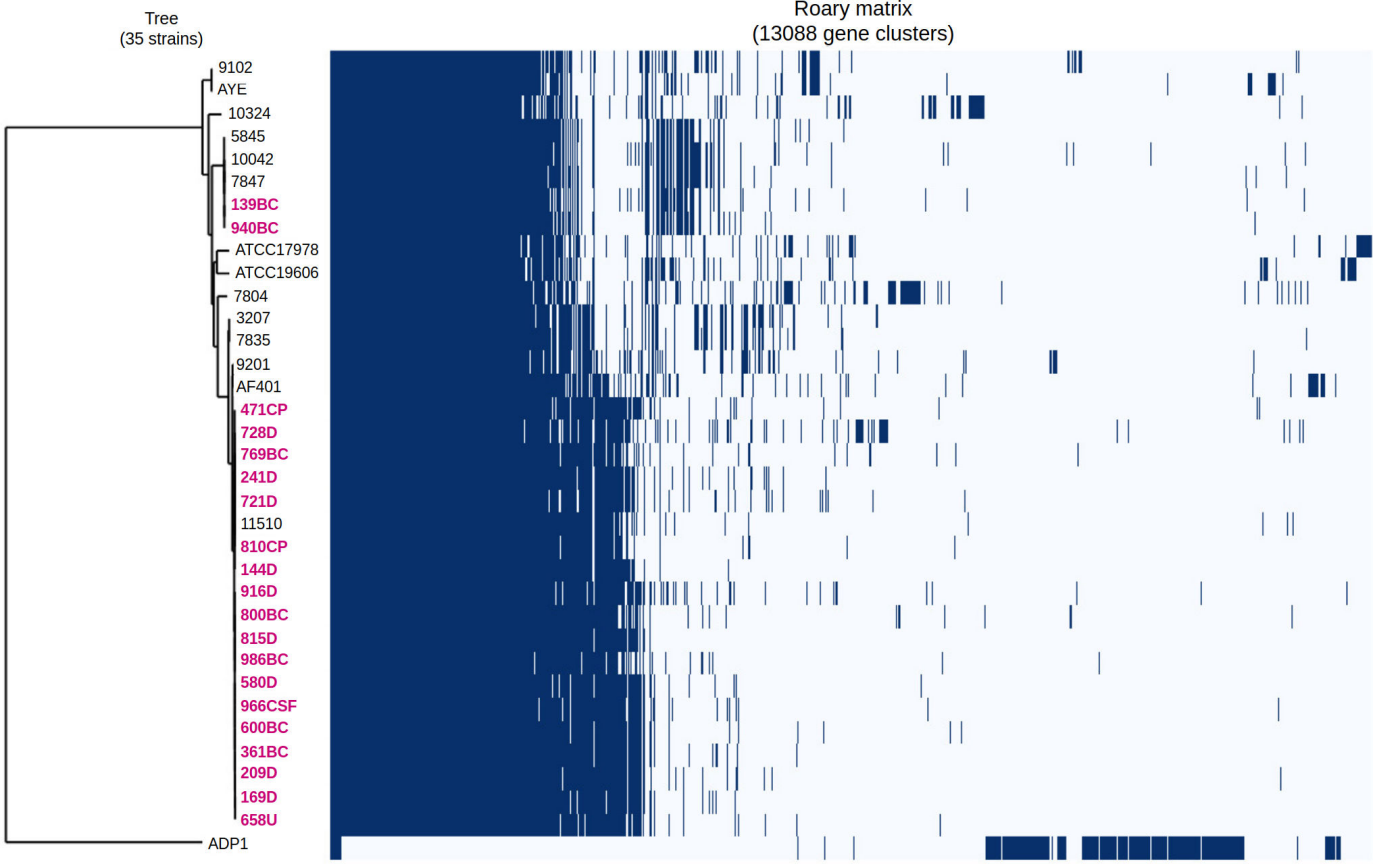
Pangenome analysis was performed for both CRAB-HIMFG and AB-DB strains. The resulting visualization depicts gene cluster presence (dark blue) and absence (light blue) of gene clusters across strains. CRAB-HIMFG strains are represented in bold pink letters. *A. balyayi* ADP1 (NCBI Accession Number: CR543861.1) was used as outgroup.

Gene clusters were classified into four categories: 54.30% (5448/10033) as cloud; 19.39% (1945/10033) as shell; 2.40% (241/10033) as soft-core; and 23.91% (2399/10033) as core (Fig. S1B; Table S2). In accordance with the pangenome analysis, a Neighbor-Joining dendrogram was constructed (Fig. 1) to illustrate the genome relatedness and the presence or absence of pangenome genes.

**Fig 2:**
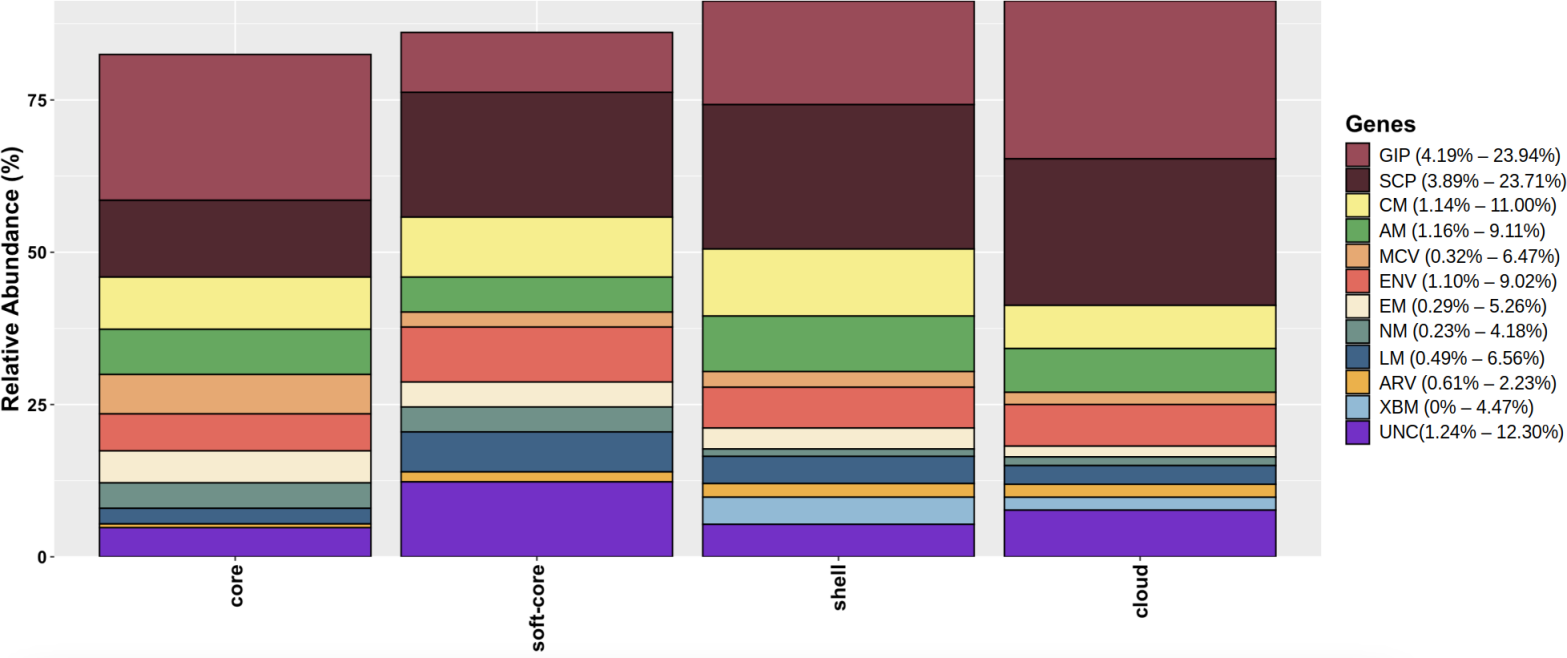
Functional annotation of each genome category was carried out using KOALA-KEGG. The bar plot displays functional characterization across *A. baumannii* genome categories (cloud, shell, soft-core, and core). The x-axis displays the functional categories, while the y-axis shows the percentage of annotated genes for the various *A. baumannii* genome categories analyzed. Color-coded bars represent distinct metabolic pathways, with complete annotation results available in Table S2. GIP, genetic information processing; SCP, signal and cellular processes; ENV, environmental information processing; CM, carbohydrate metabolism; AAM, amino acid metabolism; MCV, metabolism of cofactors and vitamins; EM, energy metabolism; NM, nucleotide metabolism; LM, lipid metabolism; XBM, xenobiotics biodegradation and metabolism; UNC, unclassified; ARV, antibiotic resistance and virulence.

To correlate the functional associations among genome categories (cloud, shell, soft-core, and core), we annotated 3,035 gene clusters using the KOALA-KEGG server (23, 24). The resulting functional classification revealed that most genes were associated with (1) genetic information processing, (2) cellular signaling and processes, and (3) metabolic pathways (carbohydrate, amino acid, lipid, and environmental metabolism). The functional distribution across all genome categories is presented as percentage values in the bar plot (Fig. 2).

Genes associated with the metabolism of cofactors, vitamins, energy, and nucleotides were predominantly found in the core and soft-core genomes. In contrast, as expected, genes related to antibiotic resistance, virulence, and the biodegradation and metabolism of xenobiotics were mainly located in the shell and cloud genomes (Table 3).

**TABLE 3:** The average percentage of each functional category across different genome types in the *A. baumannii* pangenome^*a*^^,^^*b*^

Genome	Information processing (%)			Metabolism (%)								ARV (%)
	GIP	SCP	ENV	CM	AAM	MCV	EM	NM	LM	XBM	UNC	
Core	23.94	12.61	6.07	8.56	7.42	6.47	5.26	4.18	2.56	0	4.79	0.61
Soft-core	9.84	20.49	9.02	9.84	5.74	2.46	4.10	4.10	6.56	0	12.30	1.64
Shell	17.0	23.71	6.70	11.00	9.11	2.58	3.44	1.20	4.47	4.47	5.33	2.23
Cloud	4.19	3.89	1.10	1.14	1.16	0.32	0.29	0.23	0.49	0.34	1.24	2.12

^*a*^ GIP, genetic information processing; SCP, signal and cellular processes; ENV, environmental information processing; CM, carbohydrate metabolism; AAM, amino acid metabolism; MCV, metabolism of cofactors and vitamins; EM, energy metabolism; NM, nucleotide metabolism; LM, lipid metabolism; XBM, xenobiotics biodegradation and metabolism; UNC, unclassified; ARV, antibiotic resistance and virulence.

^*b*^ Metabolic pathways with greater average representation in accessory genomes are highlighted in grey.

#### The novel sequence types identified in the CRAB-HIMFG strains

The analysis using multilocus sequence typing (MLST), based on the Pasteur (25) and Oxford (26) schemes, revealed novel STs among the CRAB-HIMFG strains, as assigned by PubMLST database. The predominant ST in the Pasteur scheme was ST^Pas^156, which corresponded to ST^Oxf^758 and ST^Oxf^1054 in the Oxford scheme (Table S1). The PubMLST database (https://pubmlst.org/organisms/acinetobacter-baumannii) currently includes over 2,500 STs in the Pasteur scheme and more than 3,000 STs in the Oxford scheme. Novel STs were identified among CRAB-HIMFG strains: ST^Pas^2796 in the Pasteur scheme, and seven in the Oxford scheme—ST^Oxf^3465, ST^Oxf^3466, ST^Oxf^3467, ST^Oxf^3468, ST^Oxf^3469, ST^Oxf^3470, and ST^Oxf^3472.

The novel strain ST^Pas^2796 associated with CRAB-HIMFG strains exhibited the following allelic profile: *cpn*60[620*], *fus*A[2], *glt*A[2], *pyr*G[2], *rec*A[29]*, rpl*B[4]*,* and *rpo*B[4], with *cpn*60 being a novel allele. The goeBURST analysis indicates that ST^Pas^2796 is a variant of ST^Pas^156, which is frequently isolated in Mexico. This approach highlights the genetic relatedness and evolutionary divergence between the STs (Fig. S2). MLST using the Oxford scheme identified six novel *gdh*B alleles and one novel *cpn*60 allele among CRAB-HIMFG strains, revealing seven novel allelic profiles (Table S3; Fig. S3). It is worth noting that the Oxford scheme is highly variable and, therefore, suitable only for fine-scale typing.

Core genome MLST (cgMLST) grouped clonal complex (CC) CRAB-HIMFG strains within cgST1332 (CC758/IC5), cgST464, and cgST6285 (CC92/IC2). Due to its higher resolution, cgMLST is considered the most suitable and comprehensive method for the epidemiological typing of *A. baumannii* (27). Lastly, the ribosomal MLST (rMLST) typing scheme proposed in 2012 (28) focuses on variation in 53 genes encoding bacterial ribosomal protein subunits. This scheme grouped CRAB-HIMFG strains in rST9802 (CC758/IC5) and rST8482 (CC92/IC2).

To assess the genetic relatedness among *A. baumannii* genomes (CRAB-HIMFG and AB-DB), we performed a core genome single-nucleotide polymorphism (cgSNP) analysis. Strikingly, the CRAB-HIMFG strains, 986BC and 815D, exhibited only one cgSNP difference, indicating they are genetically identical, even though they were obtained from different patients (P15 and P13), different wards (neurology and nephrology), different types of samples (blood culture and cholesteatoma culture), and with a gap of 29 days between their isolation. In contrast, the remaining *A. baumannii* genomes analyzed showed more than 10 cgSNP differences, indicating a more distant common ancestry (29) (Table S4).

**Fig 3:**
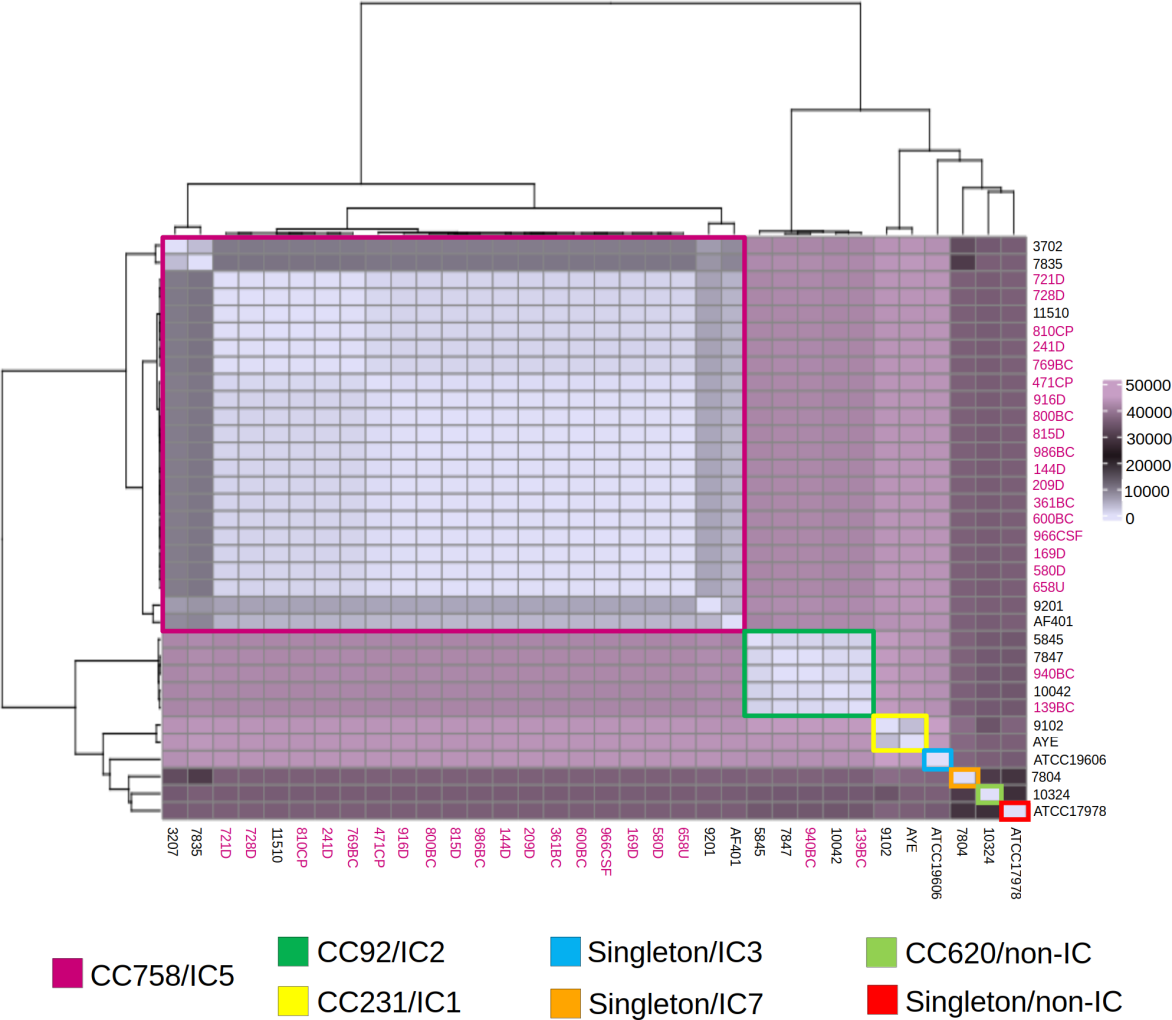
Core genome heatmap visualization of cgSNPs in *A. baumannii* strains from Mexico. The cgSNPs were calculated and compared across 20 CRAB-HIMFG strains, 11 AB-DB strains, and three *A. baumannii* reference genomes. The cgSNPs were visualized in a heatmap categorized by the seven CC/IC groupings. CRAB-HIMFG strains are highlighted in bold pink letters.

The correlations between cgSNPs, CC, and IC were analyzed using an epidemiological approach (Fig. 3). The CRAB-HIMFG strains formed a novel CC758/IC5 (pink) cluster previously classified as CC636/IC5. These strains were isolated from diverse clinical samples across Mexico over different years, including those identified as 11510, 9201, 3207, 7835, and AF401. Furthermore, CRAB-HIMFG strains 940BC and 139BC, isolated from different patients and samples in different years, clustered with strains 5845, 7847, and 10042 within CC92/IC2 (dark green). The remaining *A. baumannii* strains were clustered as follows: CC231/IC1 (yellow), which includes strains 9102 and AYE; singleton/IC7 (orange), represented by strain 7804; CC620/non-IC (light green), containing strain 10324; singleton/non-IC (red), represented by strain ATCC17978; and singleton/IC3 (turquoise), with strain ATCC19606 (Fig. 3).

#### The resistance genes *bla*A2, *mbl*, *bla*_OXA-23_, *bla*_OXA-24_, *sme*R, *sme*F, *ade*T2, *mex*T, *mdf*A, and *mdt*L were identified only in the CRAB-HIMFG strains

**Fig 4:**
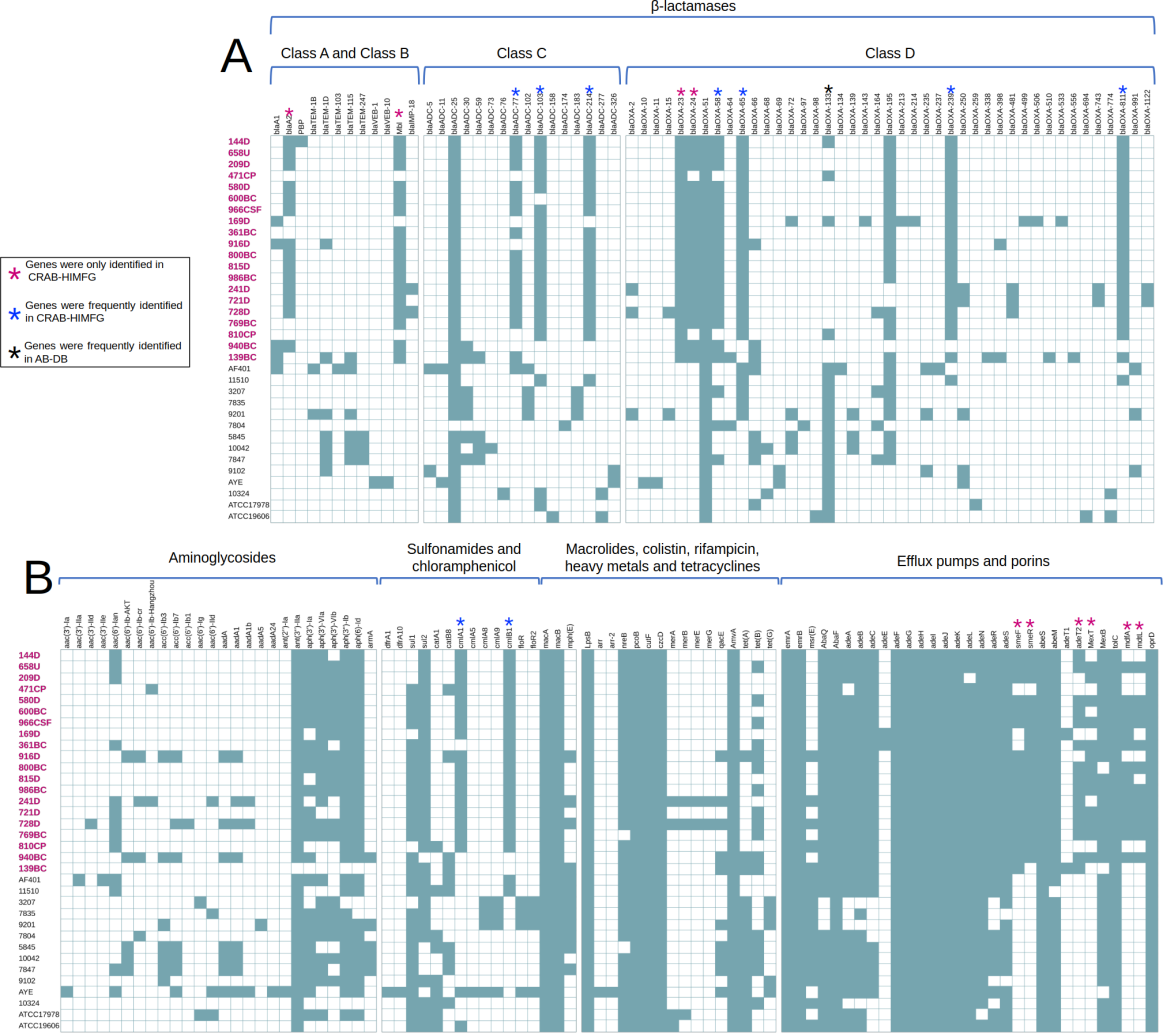
Distribution of resistance genes in the CRAB-HIMFG and AB-DB strains. The figure illustrates the presence (teal) and absence (white) of resistant genes across strains. CRAB-HIMFG strains are highlighted in bold pink text. Genes marked with a pink asterisk (*) are genes exclusive to CRAB-HIMFG strains, blue asterisk (*) genes are predominantly found in CRAB-HIMFG strains, and black asterisk (*) genes are primarily present in AB-DB strains. (**A**) β-lactamase genes. (**B**) Aminoglycoside, sulfonamides, chloramphenicol, macrolides, tetracyclines, colistin, rifampicin, efflux pumps, and porins genes.

A total of 150 resistance genes were identified in both CRAB-HIMFG and AB-DB strains with the following distribution: β-lactamases constituted 47.33% (71/150) of these resistance genes, comprising 14.08% (10/71) for class A, 2.82% (2/71) for class B, 22.54% (16/71) for class C, and 60.56% (43/71) for class D (Fig. 4A). Aminoglycoside resistance genes accounted for 17.33% (26/150) of the total, distributed as follows: 50% (13/26) for acetyltransferases (*aac*), 19.23% (5/26) for adenyltransferases (*aad*), 7.69% (2/26) for adenylation (*ant*), 19.23% (5/26) for phosphotransferases (*aph*), and 3.85% (1/26) for methyltransferases. The remaining resistance genes were distributed as follows: sulfonamide resistance accounted for 2.67% (4/150), chloramphenicol resistance 6% (9/150), macrolides and tetracyclines were observed in 2% (3/150) each, colistin and porins accounted for 0.67% (1/150), and rifampicin at 1.33% (2/150). Lastly, efflux pumps comprised 20% (30/150) of the identified resistance genes (Fig. 4B).

The analysis revealed distinct resistance gene profiles between strains. Exclusive to CRAB-HIMFG strains were *bla*A2, *mbl*, *bla*_OXA-23_, *bla*_OXA-24_, *sme*R, *sme*F, *ade*T2, *mex*T, *mdf*A, and *mdt*L (Fig. 4A and B). More prevalent in CRAB-HIMFG strains than in AB-DB strains were *bla*_ADC-77_, *bla*_ADC-103_, *bla*_ADC-214_, *bla*_OXA-58_, *bla*_OXA-65_, *bla*_OXA-239_, *bla*_OXA-811_, *cml*A1, and *cml*B1 (Fig. 4A and B). Conversely, the *bla*_OXA-133_ gene was found to be more common in AB-DB strains than in CRAB-HIMFG strains (Fig. 4A).

As expected, several intrinsic genes were identified in all CRAB-HIMFG and AB-DB strains, including *bla*_ADC-25_, *bla*_OXA-51_, *ant*(3’’)-IIa, *Lps*B, *nre*B, *pco*B, *cut*F, *amv*A, *aba*Q, *aba*F, *ade*FGH, *abe*M, and *abe*S. Additionally, *aph*(3’’)-Ib, *aph* (6)-Id, *sul*1, and *sul*2 genes were also present. For heavy metal resistance, eight genes were identified, and two genes were associated with resistance against disinfectants (Fig. 4A and B).

#### The CRAB-HIMFG and AB-DB strains displayed no differences in virulence genes in their genomes

**Fig 5:**
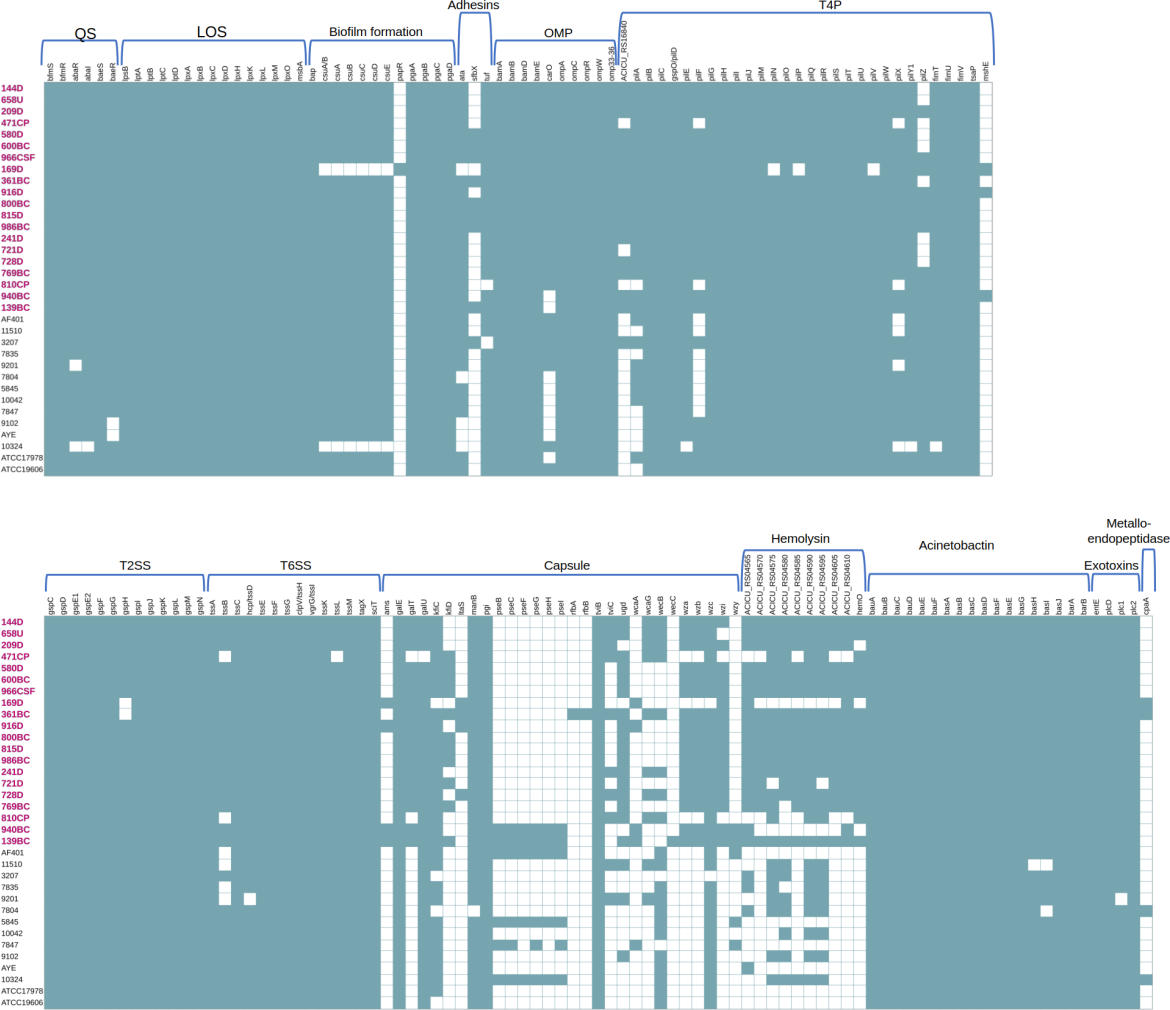
The virulence gene profiles of CRAB-HIMFG and AB-DB strains. The figure shows the presence (teal) and absence (white) of virulence genes identified across all analyzed strains in the genomes of each strain. A pink asterisk (*) marks genes exclusive to CRAB-HIMFG strains. A blue asterisk (*), genes predominantly found in CRAB-HIMFG strains, whereas those marked with a black asterisk (*) are mainly present in AB-DB strains. The names of the CRAB-HIMFG strains are highlighted in bold pink letters.

A total of 165 virulence genes were annotated, with the following distribution: 18.18% (30/165) associated with type IV pili (T4P); 17.58% (29/165) with capsule synthesis; 11.52% (19/165) with acinetobactin; 9.09% (15/165) with lipooligosaccharides (LOS); 8.48% (14/165) with the type VI secretion system (T6SS); 7.88% (13/165) with the type II secretion system (T2SS); 7.27% (12/165) with biofilm formation; 6.06% (10/165) each with hemolysin and outer membrane proteins (OMP); 3.64% (6/165) with quorum sensing (QS); 1.82% (3/165) with adhesins and exotoxins; and 0.61% (1/165) with metallo-endopeptidase (Fig. 5).

A subset of genes was identified at a lower frequency within the described virulence gene clusters. This subset included *pap*R, *sfb*X, *msh*E, *kfi*D, *lta*S, *pse*BCDFGHI, *rfb*AB, *wca*A, *wec*C, *wzy*, and *cpa*A. Some of these genes have been previously described in other bacterial genera, suggesting that these strains may have acquired them through HGT at some point (30). In contrast to the resistance genes, the virulence genes exhibited lower variability among the strains. Interestingly, although the strains carried operons for these virulence genes, not all operons were complete, as observed in examples, such as the T4P, T2SS, and T6SS.

#### KL32, KL9, and OCL10 capsule types were identified in the genomes of CRAB-HIMFG strains

The capsule is a crucial virulence factor in *A. baumannii*, acting as a barrier against environmental stress and providing an antiphagocytic effect. Among the *A. baumannii* strains studied, the distribution of capsule polysaccharide (KL) types was as follows: KL32 was found in 35.29% (12/34) of the strains; KL9 in 23.53% (8/34); and KL22 in 8.82% (3/34). Additionally, KL1, KL2, KL3, and KL23 each were found in 5.88% (2/34), while KL7, KL77, and KL195 each were identified in 2.94% (1/34). For the CRAB-HIMFG strains, the specific KL types were identified as follows: KL32 in strains 144D, 169D, 209D, 361BC, 580D, 600BC, 658U, 800BC, 815D, 916D, 966CSF, and 986BC; KL9 in strains 241D, 471CP, 721D, 728D, 769BC, and 810CP; KL2 in strain 940BC; and KL22 in strain 139BC (Table S1).

In terms of lipooligosaccharide outer core (OCL) typing, the following distribution was observed: OCL10 was present in 67.65% (23/34) of the strains; OCL1 in 20.59% (7/34); and OCL2 and OCL7 in 5.88% (2/34) each. Among CRAB-HIMFG strains, two OCL types were identified: OCL10 (present in CC758/IC5 strains) and OCL1 (found in CC92/IC2 strains) (Table S1).

#### Genomes of CRAB-HIMFG strains and insertion sequence elements played a key role in the carriage of resistance genes

**Fig 6:**
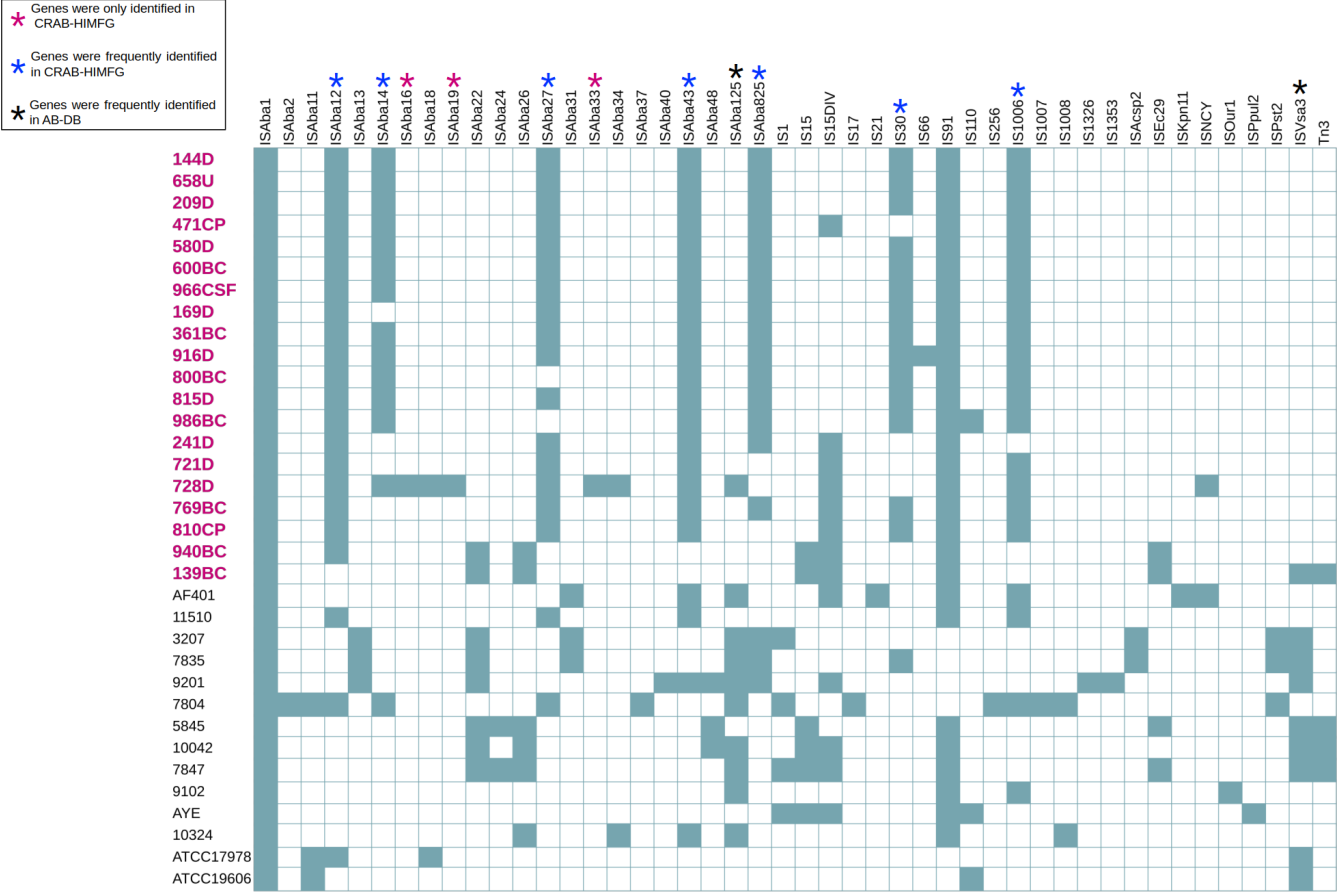
Comparison of IS element presence and absence in CRAB-HIMFG and AB-DB strains. In the figure, genes identified in the genomes of each strain are represented by a colored teal square, indicating the presence of the gene, while a white square indicates its absence. CRAB-HIMFG strains are highlighted in bold pink text. Genes marked with a pink asterisk (*) are genes exclusive to CRAB-HIMFG strains; blue asterisk (*) genes are predominantly found in CRAB-HIMFG strains; and black asterisk (*) genes are primarily present in AB-DB strains.

In the search for MGEs, we found that all 34 examined genomes (100%) contained insertion sequence (IS) elements. Among the CRAB-HIMFG strains, the most prevalent (but not exclusive) IS elements were as follows: 100% (20/20) had IS*Aba*1 and IS91; 95% (19/20) had IS*Aba*12; 90% (18/20) had IS*Aba*43; 85% (17/20) had IS1006; 80% (16/20) had IS*Aba*27; 75% (15/20) had IS*Aba*825; 70% (14/20) had IS30; and 65% (13/20) had IS*Aba*14 (Fig. 6).

**Fig 7:**
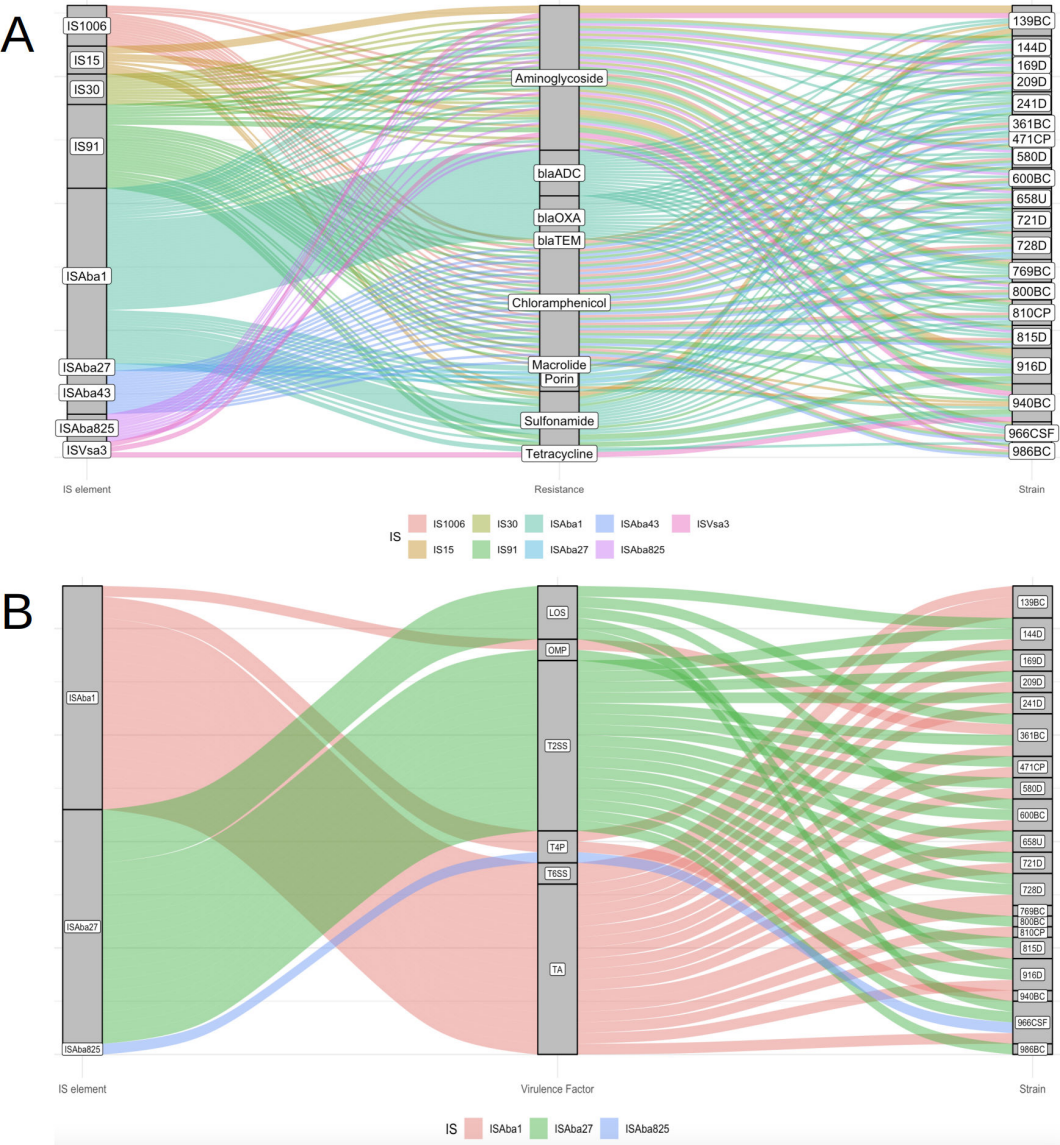
The relationship between IS elements and different gene families in CRAB-HIMFG strains. The IS element relationship between (**A**) resistance and (**B**) virulence gene families of the CRAB-HIMFG strains is represented in different colored lines.

Interestingly, resistance genes for different antibiotic classes were consistently found flanked by IS elements in the genomes of CRAB-HIMFG strains. The following associations were identified: IS*Aba*1 was linked to *bla*_OXA_, *bla*_ADC_, sulfonamide, tetracycline, aminoglycosides, and porins resistance genes; IS*Aba*27 was associated with porins; IS*Aba*43 to chloramphenicol resistance; IS*Aba*825 to aminoglycoside resistance; IS15 to porins, aminoglycoside, macrolide, and *bla*_TEM_; IS30 to aminoglycoside; IS91 to chloramphenicol, sulfonamide, and aminoglycoside; IS1006 to chloramphenicol and aminoglycoside; and IS*Vsa*3 to tetracycline and aminoglycoside resistance (Fig. 7A).

The genomic positioning of IS elements adjacent to β-lactams and aminoglycoside resistance genes was identified as follows: *bla*_ADC-5_ and *bla*_ADC-11_ genes were located downstream of two IS*Aba*1 elements; *bla*_TEM-1D_ gene was flanked between IS6 elements within the Tn3 transposon; and *bla*_ADC-73_ gene was also positioned downstream of two IS*Aba*1 elements. Finally, the *bla*_OXA-72_ gene was associated with a downstream IS*Aba*48 element, and the bla_ADC-30_ gene was located upstream of two IS*Aba*1 elements.

In IC5, the *bla*_OXA-2_ gene was flanked between IS26 (downstream) and IS1326 (upstream) elements, whereas the *bla*_ADC-25_, *bla*_ADC-30_, *bla*_ADC-214_, and *bla*_OXA-239_ genes were all located upstream of IS*Aba*1 elements.

Aminoglycoside resistance genes identified in the IC2 were linked to the following IS elements. The gene *aph*(3’)-Ia was located between IS15 elements. In several strains, including 139BC, 940BC, and 10042 (except for *aph*(3’)-Ia), the genes *aph*(6)-Id and *aph*(3’’)-Ib were found upstream of IS*Vsa*3 elements.

In strains classified under IC5, several associations between IS elements and aminoglycoside resistance were identified. The *aph*(3’’)-Ib and *aph*(6)-Id genes were located upstream of IS*Aba*1 in strains 169D, 241D, 361BC, 471CP, 721D, and 728D. The gene *aph*(3’)-VIa was located between a pair of IS30 and IS*Aba*825 elements in strains 209D, 800BC, and 986BC. The gene *aph*(3’)-Ia was flanked by two IS15 elements. The gene *aph*A6 was located between IS30 and IS*Aba*825. Meanwhile, the gene *aac*(6)-Ian was found between IS91 and IS6 elements in strains 241D, 721D, 728D, and 769BC. The gene *aac*(6’)-Ib’ was upstream of IS6, and the gene *aad*A was found downstream of IS91 in strain 916D (Table S5).

Three IS elements were identified flanking genes encoding key virulence factors. The IS*Aba*1 was located adjacent to genes involved in T4P, T6SS, OMP, and toxin-antitoxin (TA) systems. In contrast, IS*Aba*27 flanked genes encoding LOS and OMP genes, while IS*Aba*825 was flanked by T4P genes (Fig. 7B). On the other hand, the TA systems were found downstream of IS*Aba*1. Genes for T2SS were located downstream of a pair of IS*Aba*27 and IS30 elements, while the gene *lpx*O was situated upstream of two IS*Aba*27 elements.

IS elements were identified upstream and downstream of ABC transporters, including *ton*B, ABC transporters (IS*Aba*1), and MFS transporters (IS15). The membrane protein *omp*W was also located downstream of IS*Aba*125, while *opr*D was located upstream of IS5-family elements. Additionally, the assembly of T4P was identified upstream of the IS*Aba*825 elements. In strain 916D, two pairs of *bap* genes were identified downstream of a pair of IS15 elements (Table S5).

#### Plasmid analysis identified the R1, R3, and RP families

The plasmid analysis of the CRAB-HIMFG and AB-DB strains demonstrated remarkable genetic diversity. We identified 72 plasmids distributed among the analyzed strains. Classification based on replication (*rep*) genes revealed the following distribution:

- Major plasmid types: 23.61% (17/72) were categorized as R3-T26; 22.22% (16/72) *rep*-less (replication-less); and 15.28% (11/72) as R1-T1.
- Intermediate frequency types: 6.94% (5/72) were classified as R3-T26/R3-T39 and RP-T1 each, while 5.55% (4/72) were categorized as R3-T17.
- Less frequent types: 4.17% (3/72) were R3-T8; 2.78% (2/72) were R3-T5; and 1.39% (1/72) each for R3-T1, R3-T3, R3-T4/R3-T13, R3-T7, R3-T12, R3-T18, R3-T33/R3-T13, R3-T57, and non-typeable. Notably, 9.72% (7/72) of the plasmids carried multiple *rep* genes (multi-*rep* plasmids) (Fig. S4), consistent with previous reports (31).

In the CRAB-HIMFG strains, 48 plasmids averaging three plasmids per strain were identified, with the following distribution: 33.33% (16/48) classified as R3-T26; 31.25% (15/48) as *rep-*less; 20.83% (10/48) as R1-T1; 10.42% (5/48) as R3-T26/R3-T39; 2.08% (1/48) as R3-T33/R3-T13; and non-typeable, each. Three plasmid types (R3-T26/R3-T39, R3-T33/R3-T13, and non-typeable) demonstrated exclusive association with CRAB-HIMFG strains (Table S6).

Additionally, CRAB-HIMFG plasmids were typically characterized by the absence of genes encoding virulence factors, stress response mechanisms, or resistance genes. Instead, they primarily encode two copies of IS*Aba*27. However, there were significant exceptions among CRAB-HIMFG plasmids. For instance, plasmid pAba986BC stands out by incorporating genes that encode for T4P assembly, the *hok*/*sok* TA system, and the IS30 and IS110 elements. In contrast, plasmid pAba728Dc emerges as the most genetically diverse, boasting the highest number of resistance genes, which include *bla*_OXA-2_, *bla*_OXA-58_, *msr*(E), *mph*(E), *sul*1, *sul*2, *cml*A, *aph*(3’)-VIa, *bla*_IMP-18_, *aac*(6’)-Ib, *aad*A1, *qac*EΔ1, *aac*(3)-IId, *aph*(3’’)-Ib, *aph*(6)-Id, and *mer*ABGE.

Furthermore, the plasmid pAba728Dc encodes essential genes involved in the assembly of T4P and type IV secretion system (T4SS), as well as genes related to stress response, including *knr4*/*smi1*-like and *csd*. This plasmid was characterized by two distinct TA systems (*brn*T/*brn*A and *hic*A/*hic*B) and possesses the highest number of recorded IS elements, including IS*Aba*1, IS*Aba*14, IS*Aba*16, IS*Aba*19, IS*Aba*33, IS*Aba*34, IS*Aba*125, IS1006, and ISNCY (Table S6). This comprehensive analysis underscores the complex plasmid architecture within these clinical strains, revealing substantial genetic variability that may contribute to their adaptability and resistance profiles, providing critical insights into their genetic architecture.

The CRAB-HIMFG plasmids that carried the multi-*rep* T3-T26/T3-T39, specifically pAba209Db, pAba580Dc, pAba769BCc, pAba169Da, and pAba721Dc, showed significant differences compared to the T3-T26 plasmids. These plasmids were nearly double the molecular size of the T3-T26 plasmids and contained two copies of each gene, which increases their genetic complexity. Additionally, the T3-T26/T3-T39 plasmids did not share the same molecular size (Table S6). Outstandingly, the genes encoding for antibiotic resistance were mainly located on chromosomes rather than plasmids.

### Phenotypic analysis

#### CRAB-HIMFG strains were classified as MDR-6

The antibiotic susceptibility testing revealed that 100% (20/20) of the CRAB-HIMFG strains were classified as MDR. The strains exhibited resistance to the following antibiotics:

- Penicillins: PRL (100%, 20/20)
- β-lactams combined with inhibitors: SAM 25% (5/20) and TZP 100% (20/20)
- Third- and fourth-generation cephalosporins: CAZ 95% (19/20), FEP 95% (19/20), and CRO 90% (18/20)
- Carbapenems: IPM 100% (20/20)
- Aminoglycosides: GEN 5% (1/20)
- Fluoroquinolones: CIP 95% (19/20)
- Antagonists of the folate pathway: SXT 90% (18/20)

All strains 100% (20/20) were found to be susceptible to tigecycline (TG) and intermediate to colistin (COL) (Table S7). Based on the number of resistant antibiotics, 85% (17/20) of the strains were classified as MDR-6, while the remaining 15% (3/20) were classified as MDR-3, MDR-5, and MDR-7 in 5% (1/20) (Table S7).

#### One-half of the CRAB-HIMFG strains were high biofilm formers

**Fig 8:**
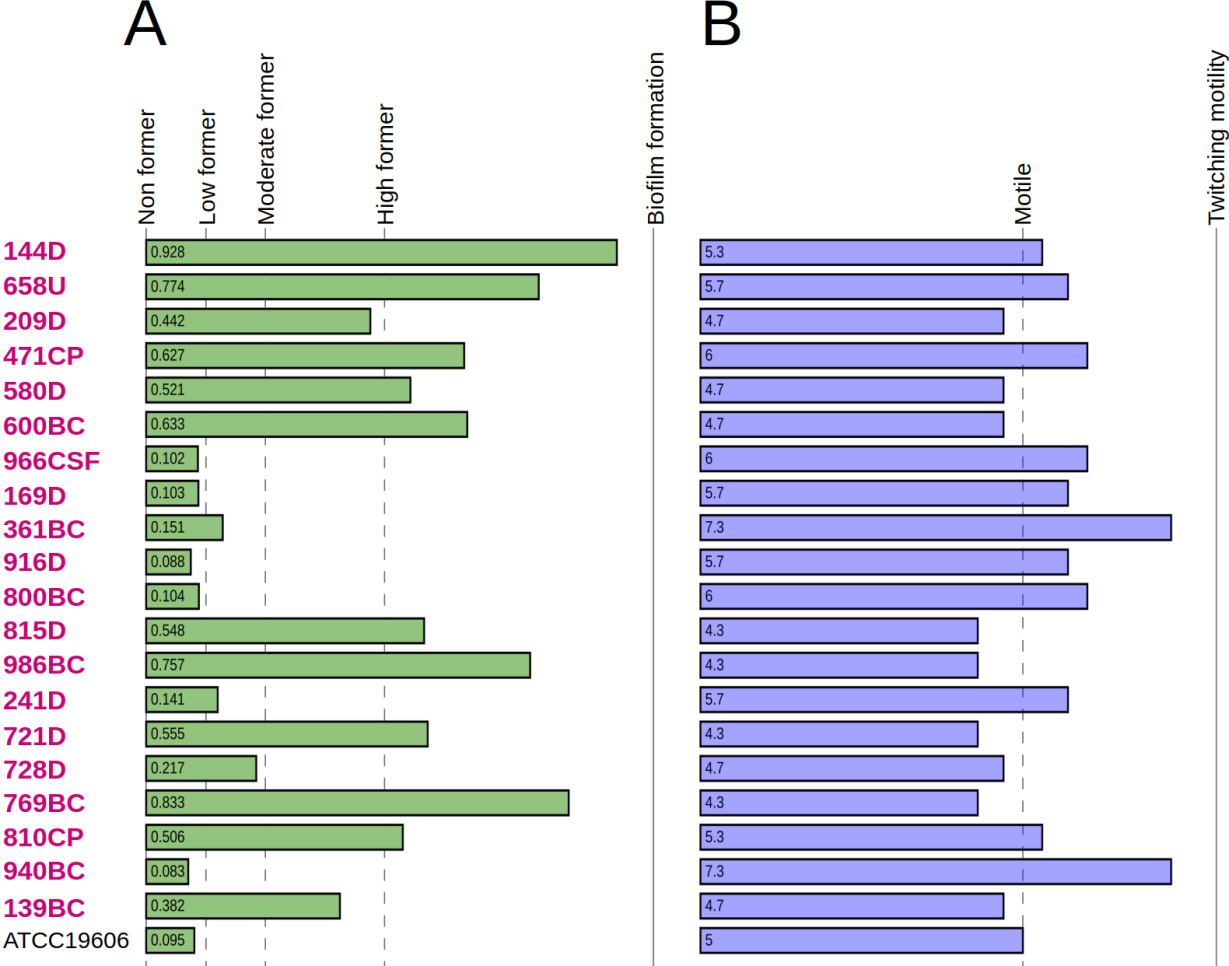
Biofilm formation and twitching motility assays of CRAB-HIMFG strains. The *in vitro* assays of biofilm formation (**A**) are shown in green bars. The cutoffs of non, low, moderate, and high formers are represented as dashed lines. Twitching motility (**B**) assays are shown in purple bars with the cutoff of positive motility represented as a dashed line.

Biofilm formation was assessed by measuring absorbance against a negative control cutoff (Ac), which was determined to be Ac = 0.118. Strains were categorized based on their absorbance values as follows: non-biofilm formers (≤0.118), low biofilm formers (0.119–0.234), moderate biofilm formers (0.235–0.469), and high biofilm formers (≥0.470). Out of the 20 CRAB-HIMFG strains tested, 50% (10/20) were classified as high biofilm formers, 10% (2/20) as moderate biofilm formers, 15% (3/20) as low biofilm formers, and 25% (5/20) as non-biofilm formers (Fig. 8A).

#### CRAB-HIMFG strains exhibited twitching motility.

*A. baumannii* strains do not possess flagella. However, they exhibit twitching motility mediated by T4P, which facilitates their spread on abiotic surfaces (32). This type of motility has been linked to the ability of these strains to cause systemic infections. In twitching motility assays, the classification of CRAB-HIMFG strains showed that 55% (11/20) displayed twitching motility, while 45% (9/20) did not. Among the strains exhibiting twitching motility, 36.36% (4/11) were isolated from various fluid samples, including bronchial aspirates and catheters. Additionally, 27.27% (3/11) were obtained from blood cultures, 18.18% (2/11) from stool cultures, and 9.09% (1/11) from urine cultures and cerebrospinal fluid (Fig. 8B).

## DISCUSSION

The escalating antibiotic resistance in *A. baumannii* represents a significant public health challenge. Carbapenem resistance rates are reaching 39% in clinical settings with a mortality rate as high as 91% (1, 33, 34). This MDR pathogen also shows concerning resistance patterns to fluoroquinolones (50% to 73%) and aminoglycosides (19% to 31%). Our study provides crucial insights into the genomic epidemiology of CRAB infections, with particular focus on the understudied pediatric population, while revealing novel resistance mechanisms and virulence determinants in Mexican clinical isolates.

The CRAB-HIMFG strains analyzed in this work exhibited distinct clinical and molecular characteristics that set them apart from documented populations. While prior studies describe ventilator-associated pneumonia primarily in male patients aged 1 to 5 years and neonates, with mortality rates ranging from 30.8% to 42.9% (35, 36), our CRAB-HIMFG cohort showed bloodstream infections predominating in pediatric patients with ALL2.

Molecular characterization revealed two dominant ICs: IC2, a globally distributed carbapenem-resistant lineage, and IC5, reported primarily in North America, Brazil, and Bolivia (37). The prevalence of IC5, represented by ST^Pas^156 in Mexican hospitals, is a significant concern due to the high-risk MDR nature of strains (19, 37, 38). Notably, ST^Pas^156 has been previously linked to ST^Oxf^758, which was isolated from five Mexican hospitals (19, 37–39), and to ST^Oxf^1054, which was identified in strains from Mexico City (39). Comparison of the two MLST schemes reveals that the Pasteur scheme was more effective for typing *A. baumannii* strains, with a greater number of well-defined STs than the Oxford scheme (40–43).

We observed an association between IS elements and clinically significant genes, including β-lactamases class A (*bla*_TEM-1D_), class C (*bla*_ADC-25_ and *bla*_ADC-30_) (19, 44), and class D (*bla*_OXA-239_) (38). Otherwise, our findings revealed the presence of genes that have not been reported previously and lacked any association with IS elements (*bla*_TEM-115_, *bla*_TEM-247_, *bla*_ADC-59_, *bla*_ADC-103_, *bla*_OXA-195_, *bla*_OXA-338_, *bla*_OXA-556_, and *bla*_OXA-811_). Finally, we identified strain 139BC, which carries the *bla*_OXA-510_ gene, a novel variant of *bla*_OXA-51_ previously detected in a carbapenem-sensitive strain (45).

Key virulence factors identified include capsule types OCL10, KL32, and KL9 in IC5 strains, as well as OCL1 in IC2, along with secretion system machinery (T2SS and T6SS) and acinetobactin synthesis genes. Currently, there is limited information regarding IS elements located upstream or downstream of virulence factors (46), making this study one of the first of its kind.

Plasmid analysis uncovered significant findings, including the first reported R1-family plasmids in IC5 strains, as well as 12 out of 69 types of R3-family plasmids. Additionally, we found RP-family plasmids in IC5 carrying *aph*(3’)-VIa gene. Finally, a *rep*-less family was also identified. These plasmids do not have identifiable replication initiation proteins, suggesting the presence of an alternative replication mechanism (47). The identification of various systems within CRAB-HIMFG plasmids underscores their role in environmental adaptation and the dissemination of genes. This includes type II TA systems, such as *rel*E/*rel*B and *brn*T/*brn*A (48–50); type I TA systems, like *hok*/*sok* (51); conjugation machinery, which encompasses T4P and T4SS (52–55); and the zinc transportation system *znu*D2 (56). Furthermore, adaptation to environmental stress was linked to *knr*4/*smi*1-like (57) in CRAB-HIMFG plasmids, highlighting their role in environmental adaptation and gene dissemination. The presence of IS*Aba*27 in *rep*-less plasmids raises particular concern for future resistance gene acquisition.

In AB-DB plasmids, several stress-related genes were identified, including *dksA* and *uspA* (58–60). The gene *uvrA* is important for repairing DNA damage caused by UV irradiation (61). In contrast, the gene *ohr* is recognized for its role in conferring resistance to fatty acid hydroperoxides and peroxynitrite compounds (62).

Phenotypic characterization confirmed the clinical relevance of these genomic findings. All strains exhibit an MDR profile, with 50% demonstrating high biofilm formation and over half showing twitching motility. These traits contribute to enhanced persistence in hospital environments and increase pathogenic potential (3).

This work significantly enhances our understanding of CRAB genomics in several important and novel ways. First, it establishes the molecular epidemiology of pediatric CRAB infections in Mexico, highlighting differences from global patterns. Second, it uncovers novel STs and resistance variants broadening known diversity. Finally, it provides the first comprehensive analysis of plasmid content in Mexican strains, identifying unique genetic features contributing to their success as hospital pathogens.

The clinical implications of these findings are significant, highlighting the need for tailored infection control measures. Continuous surveillance is essential for monitoring the dissemination of novel STs and plasmid-borne resistance determinants.

### Conclusion

CRAB exhibits extraordinary genomic plasticity, continuously acquiring resistance and virulence genes through horizontal gene transfer. Our study reveals diverse clonal populations within a single hospital, including novel STs, making a significant contribution to the field of bacterial genomics. In addition to resistance genes, CRAB harbors plasmids encoding virulence factors, toxin-antitoxin systems, and mechanisms for maintaining these traits, which aid in clinical settings. These findings highlight genomic complexity of CRAB, particularly in pediatric infections, where surveillance remains limited. This study provides valuable insights into CRAB’s dynamics, emphasizing the importance of genomic surveillance to guide infection control and treatment strategies.

## MATERIALS AND METHODS

### Genomic analysis

#### Carbapenem-resistant *A. baumannii* HIMFG strains

This study focused on 20 strains of carbapenem-resistant *A. baumannii* (CRAB), which were isolated from 15 patients at HIMFG between 2015 and 2017. The strains were selected based on their carbapenem resistance profile. The production of carbapenemases was confirmed using the Rapidec Carba NP test (BioMérieux, Marcy l’Étoile, France).

Furthermore, 11 complete genomes of *A. baumannii* strains from Mexico, available in the BV-BRC database, were included (21). Additionally, three genomes from reference strains of *A. baumannii* were included (Table S1).

#### Genomic DNA extraction and purification

CRAB-HIMFG genomic DNA was extracted and purified using the phenol-chloroform method (63). Following extraction, the DNA was subjected to advanced sequencing utilizing the Illumina NextSeq500 platform, generating 2 × 75 bp paired-end reads at a depth of 40×. Additionally, sequencing was performed using Oxford-Nanopore MinION technology, achieving a depth of 50×. Both sequencing approaches were performed at the Unidad Universitaria de Secuenciación Masiva y Bioinformática (UUSMB) within the Instituto de Biotecnología, UNAM.

#### Quality, assembly, and annotation of genomes

The quality of the reads from the Illumina NextSeq500 and Oxford-Nanopore MinION was assessed using FastQC v0.11.9 (64). The Illumina NextSeq 500 reads were trimmed with Trimmomatic v0.39 (65), while the Oxford Nanopore MinION reads were processed with NanoFilt v2.8.0 (66). Genome assembly was carried out with Flye 2.9.2-b1786 (67) and Unicycler v0.5.0 (68). The quality of assembly was evaluated using QUAST v5.2.0 (69), completeness was assessed with Bandage v0.8.1 (70), while both completeness and contamination detection were performed using CheckM v1.2.4 (71). Genome annotation was performed with Prokka v1.14.6 (72) and Bakta Web (https://bakta.computational.bio/)(73). The JSpeciesWS (https://jspecies.ribohost.com/jspeciesws/) server (74), based on ANIb and ANIm algorithms, was used to determine the ANI. TETRA was also used.

The sequenced strains were registered at NCBI BioProject PRJNA1258274, and their accession numbers are mentioned in Table S1.

#### Pangenome and core genome analysis

Pangenome analysis was conducted using Roary v3.11.2 (22). An initial analysis was performed using only *A. baumannii* genomes to cluster genes into core, soft-core, shell, and cloud categories. Subsequently, a second run was conducted to construct a Neighbor-Joining dendrogram based on the core gene alignment generated with MAFFT and rooted using *Acinetobacter balyayi* ADP1 (NCBI Accession Number: CR543861.1) as outgroup. The cgSNPs were identified using Snippy v4.6.0 (75), and non-recombinant genes were determined by PhiPack v1.1.4 (76). A SNP distance matrix was then created with snp-dists v0.8.2 (77) and visualized as a heatmap with R v4.3.1 packages.

### MLST, cgMLST, and rMLST typing

MLST was conducted *in silico* using two different schemes: the Oxford scheme (26), which includes the *glt*A, *gyr*B, *gdh*B, *rec*A, *cpn*60, *gpi*, and *rpo*D genes; and the Pasteur scheme (25), which consists of *cpn*60, *fus*A, *glt*A, *pyr*G, *rec*A, *rpl*B, and *rpo*B genes. The goeBURST analysis with PHYLOViZ v2.0 (78) was used to visualize the ST relationships among strains, including both newly assigned PubMLST STs and those previously registered. Additionally, cgMLST (27) and rMLST (28) were performed to classify samples based on 2,390 genes and 53 genes encoding bacterial ribosomal protein subunits, respectively, using the PubMLST database.

### Ortholog gene annotation

The ortholog gene annotation was conducted thoroughly to categorize genes from core, soft-core, shell, and cloud genomes using BlastKOALA, following the established guidelines outlined in (https://www.kegg.jp/blastkoala/) (23). To better understand the connections to metabolic pathways, the annotated amino acid sequences were analyzed using the KEGG server, as described in (https://www.kegg.jp/kegg/) (24). This comprehensive approach provides a solid understanding of gene functions within metabolic frameworks.

### Capsule typing

The KL and OCL were typed *in silico* with the Kaptive server(https://kaptive-web.erc.monash.edu/jobs) (79), referencing the *A. baumannii* KL and OCL databases and the AYE strain.

### Resistance and virulence gene annotation

Resistance genes were identified by BLAST v2.2.8 (80) against the Comprehensive Antibiotic Resistance Database (CARD) v4.0.0 (81) and ResFinder v4.1 (82), and virulence genes were identified using the Virulence Factor Database (VFDB) (https://www.mgc.ac.cn/VFs/) (83). A sequence identity and coverage threshold of ≥90–95% was established.

### Plasmid and mobile genetic elements identification

The plasmid assembly and identification were realized using Plassembler v1.6.2 (84), with reads from both Illumina NextSeq 500 and Oxford Nanopore MinION. The predicted plasmids were confirmed through annotation with Bakta Web (73), focusing on key features such as origins of replication and mobilization genes (31). To ensure comprehensive classification, the plasmids were typed according to the *A. baumannii* plasmid typing database v3.0 available at (https://github.com/MehradHamidian/AcinetobacterPlasmidTyping) (85). The maximum likelihood (ML) pangenomic phylogeny for plasmids was executed using Get_Homologues v07.11.2023 (86) and Get_Phylomarkers v2.5.0_2023-01-14 (87).

In this study, an in-depth analysis of other MGE within the *A. baumannii* genomes, in addition to plasmids, was also conducted. IS and transposons were systematically identified according to the Bakta Web (73) annotation and were confirmed using their respective databases: ISFinder for insertion sequences (https://isfinder.biotoul.fr/) (88) and TnCentral for transposons (https://tncentral.ncc.unesp.br/) (89). This comprehensive study highlights the complexity and adaptability of the genetic structure of *A. baumannii*.

### Phenotypic assays

Phenotypic assays were performed exclusively on the CRAB-HIMFG strains.

### Minimal inhibitory concentration assay

The antibiotic-resistant profiles were previously reported (5). The methodology adhered to the guidelines set forth by the Clinical and Laboratory Standards Institute (CLSI, 2024) (90), where susceptibility testing for TG and COL (Sigma-Aldrich, MO, USA) was set using the microdilution method in Mueller–Hinton cation-adjusted broth (MHCAB, BD-Difco, MD, USA). MDR strains were defined as those with acquired non-susceptibility to at least one antibiotic in three or more categories (91).

### Biofilm formation assay

Biofilm formation was assessed using the crystal violet (CV) staining method (92). *A. baumannii* colonies were grown overnight and then suspended in brain heart infusion broth (BHI, BD-Difco, Franklin Lakes, NJ, USA), adjusting the concentration to 0.5 MacFarland (1 × 10^8^ cells/mL). The cultures were incubated at 37°C for 48 h under static conditions. The samples were fixed in 2% formalin. Afterward, 2% of CV was added to the wells, and the samples were incubated for 20 min at room temperature and washed three times with PBS before adding ethanol (100%) for 20 min. The assays were completed in triplicate in 96-well microplates. Biofilm formation was measured on a spectrophotometer (MultiskanTM FC, ThermoFisher Scientific Inc., Waltham, MA, USA) at 620 nm. The mean (A) and standard deviation (SD) were calculated for each assay. The Ac was defined as the value of three times the SD above the A of the negative control (Ac = A of the negative control + 3 SD of the negative control). The strains were classified into four categories based on the Ac and the A: no producers (A ≤ Ac), low producers (Ac <A ≤ 2 Ac), moderate producers (2 Ac <A ≤ 4 Ac), and high producers (4 Ac <A).

### Twitching motility assay

Twitching motility was assessed using the subsurface agar method in Pleuropneumonia-like organism media (PPLO, BD-Difco, Franklin Lakes, NJ, USA) containing 1% agar (93). Freshly grown cultures of *A. baumannii* were inoculated into the agar, stabbing to facilitate the spread of bacteria at the interface between the bottom of the Petri dish and the agar layer. The plates were incubated at 37°C for 24 h. Positive twitching was defined as cultures exhibiting a zone diameter greater than 5 mm. Each assay was executed three times for each strain.

## Data Availability

Raw data have been deposited in the National Center for Biotechnology Information and https://github.com/Dabiguina94/CRAB-HIMFG. The accession numbers of the genomes and plasmids can be found in the GitHub repository. The sequenced strains were registered at NCBI BioProject PRJNA1258274, and their accession numbers are mentioned in Table S1.

## SUPPLEMENTAL MATERIAL

Supplemental figures spectrum.01676-25-s0001.pdf Fig. S1 to S4.

Supplemental tables spectrum.01676-25-s0002.xlsx Tables S1 to S7.

ASM does not own the copyrights to Supplemental Material that may be linked to, or accessed through, an article. The authors have granted ASM a non-exclusive, world-wide license to publish the Supplemental Material files. Please contact the corresponding author directly for reuse.
