# Supplementary material for: Comparative genomics of carbapenem-resistant Acinetobacter baumannii isolated from pediatric patients in a tertiary care hospital
Source: Microbiol Spectr. 2025 Oct 6;13(11):e01676-25. doi: 10.1128/spectrum.01676-25 (PMC12584756; doi:10.1128/spectrum.01676-25)
Supplement: Supplemental figures — Fig. S1 to S4. [file spectrum.01676-25-s0001.pdf]

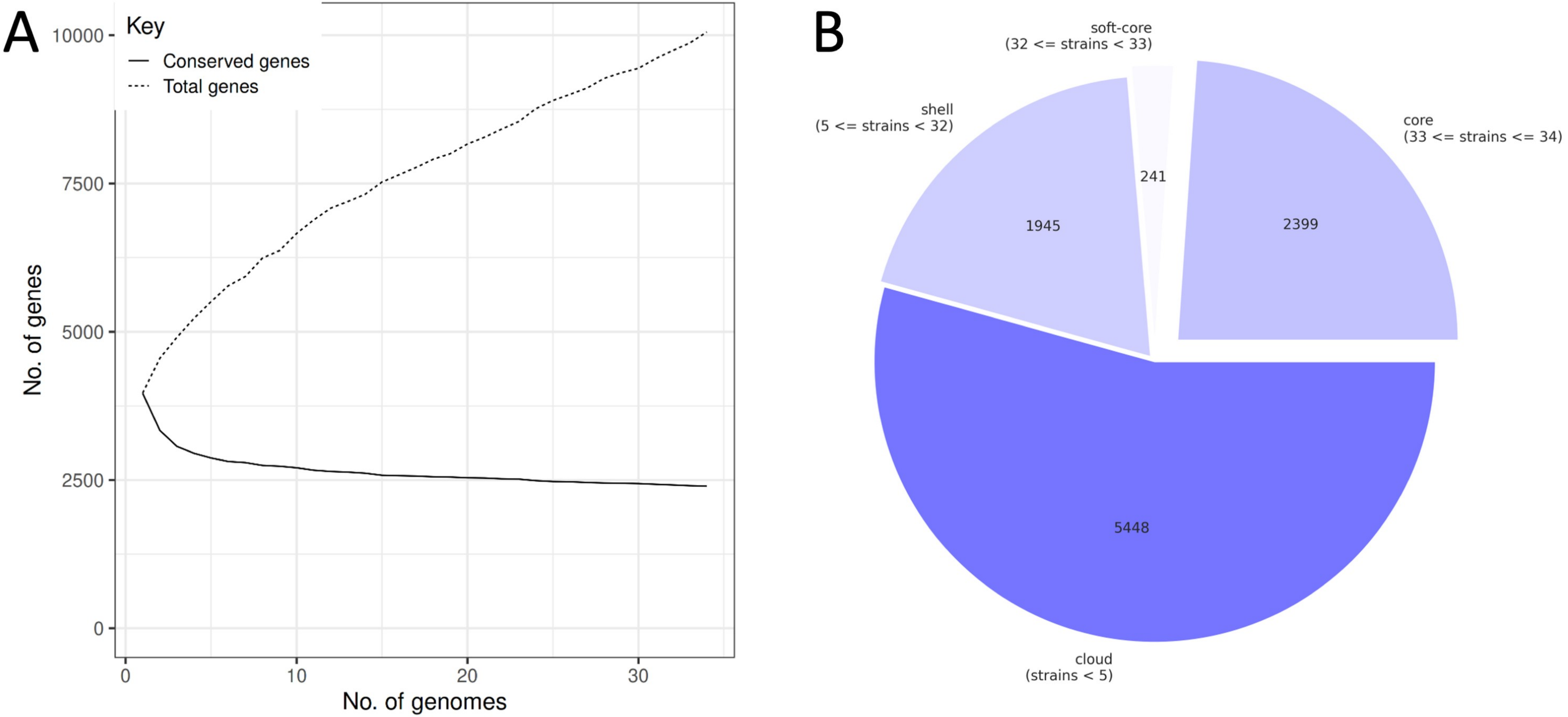

FIG S1 Analysis of the Pangenome and Core Genome CRAB-HIMFG and AB-DB. A) Pangenome dynamics showing a total of gene repertoire expansion (dotted line, representing new gene acquisition) and core genome reduction (solid line, representing conserved genes). B) Pangenome composition depicted as a pie chart, with gene clusters categorized as: cloud (54.30%), shell (19.39%), soft-core (2.40%), and core (23.91%) genomes.

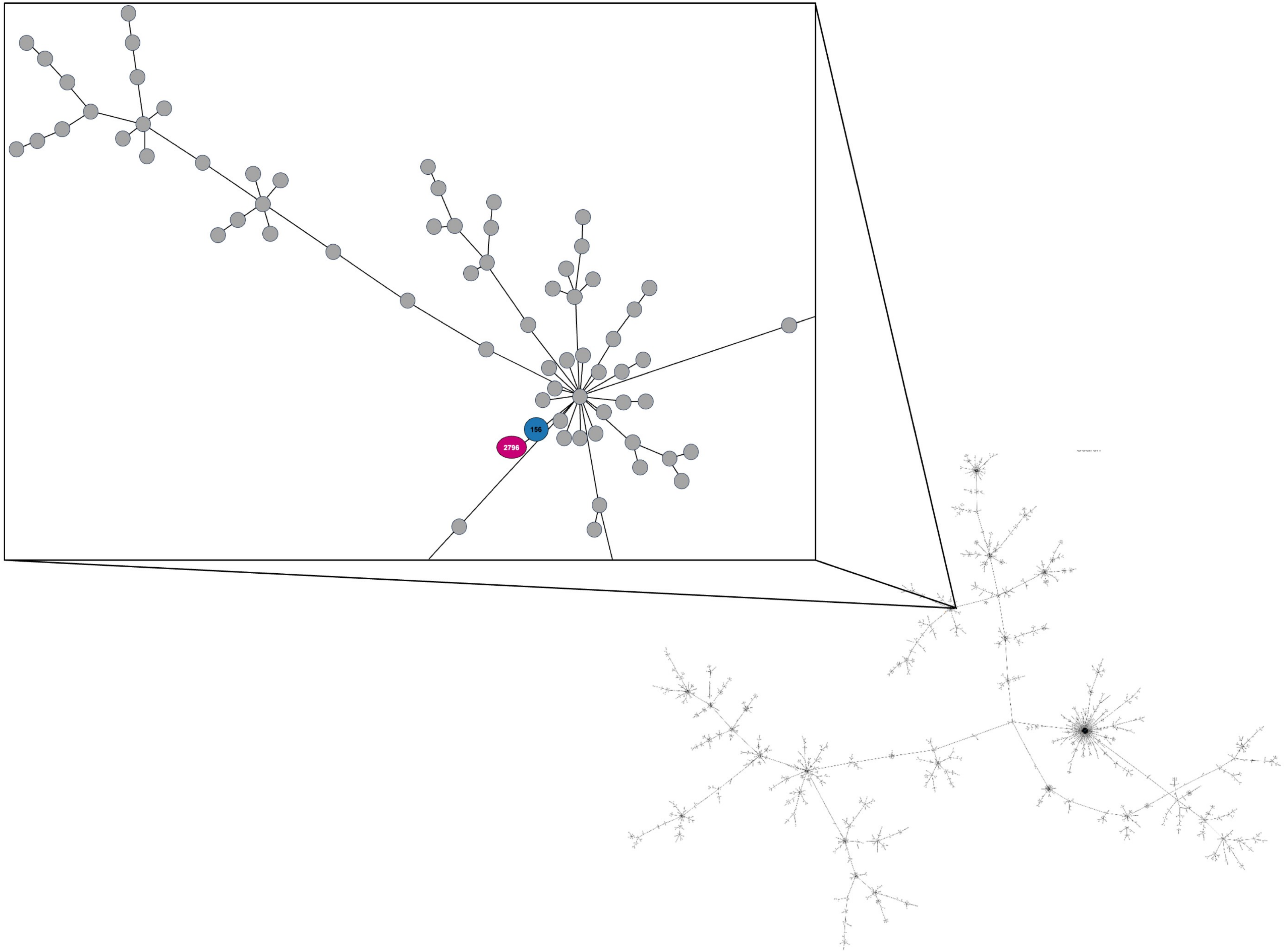

FIG S2 goeBURST analysis of the MLST Pasteur scheme for CRAB-HIMFG strains reveals that the novel ST^{Pas}2796 (pink dot), is a variant of ST^{Pas}156 (blue dot).

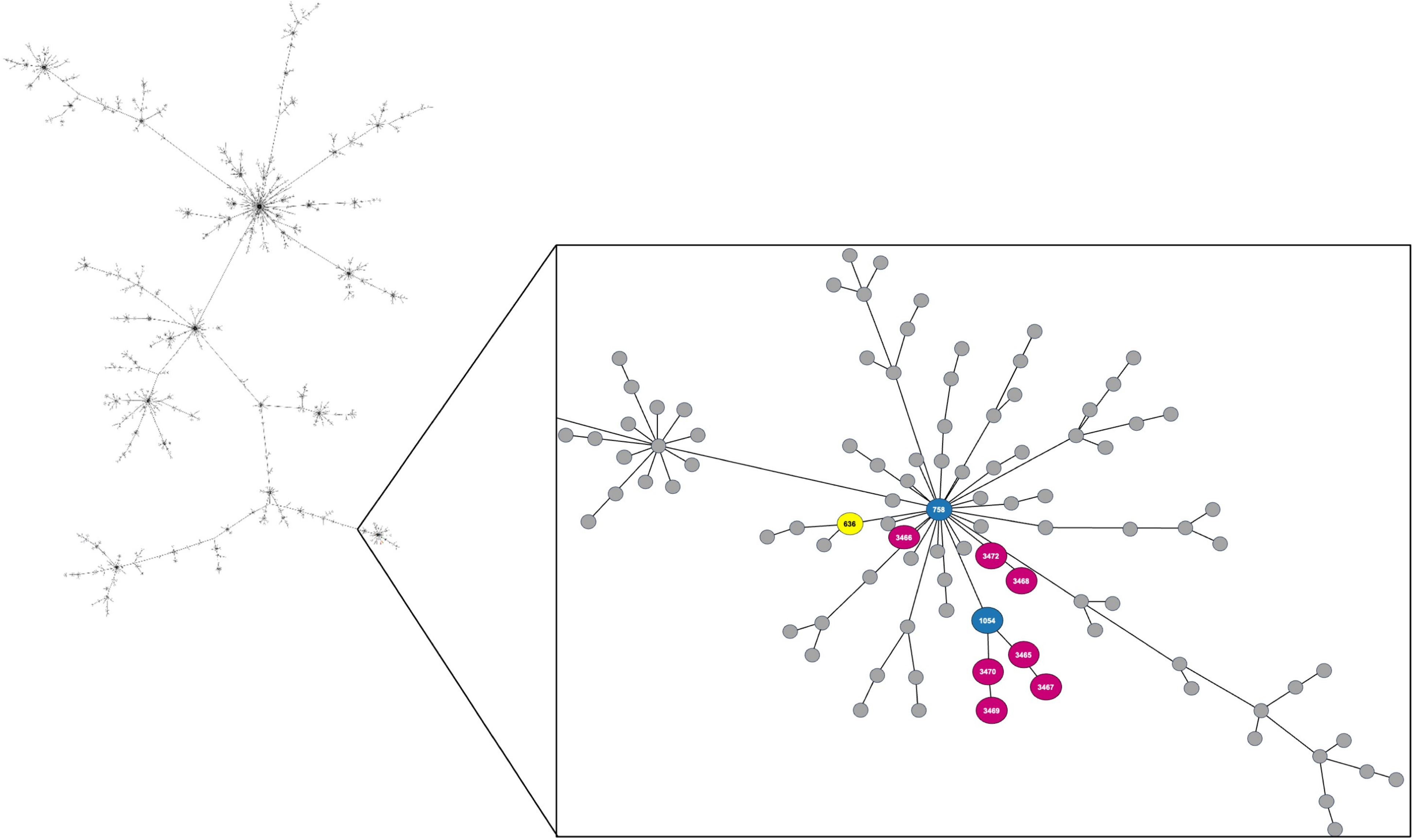

FIG S3 goeBURST analysis of the novel STs in the Oxford MLST scheme for CRAB-HIMFG strains. The zoomed-in view highlights novel STsOxf variants (blue dots) derived from STOxf758 and STOxf1054. This analysis reveals the emergence of CC758 as a new CC. Novel CRAB-HIMFG STs are indicated by pink dots while the previously described STOxf636 (yellow dot) is shown for reference.

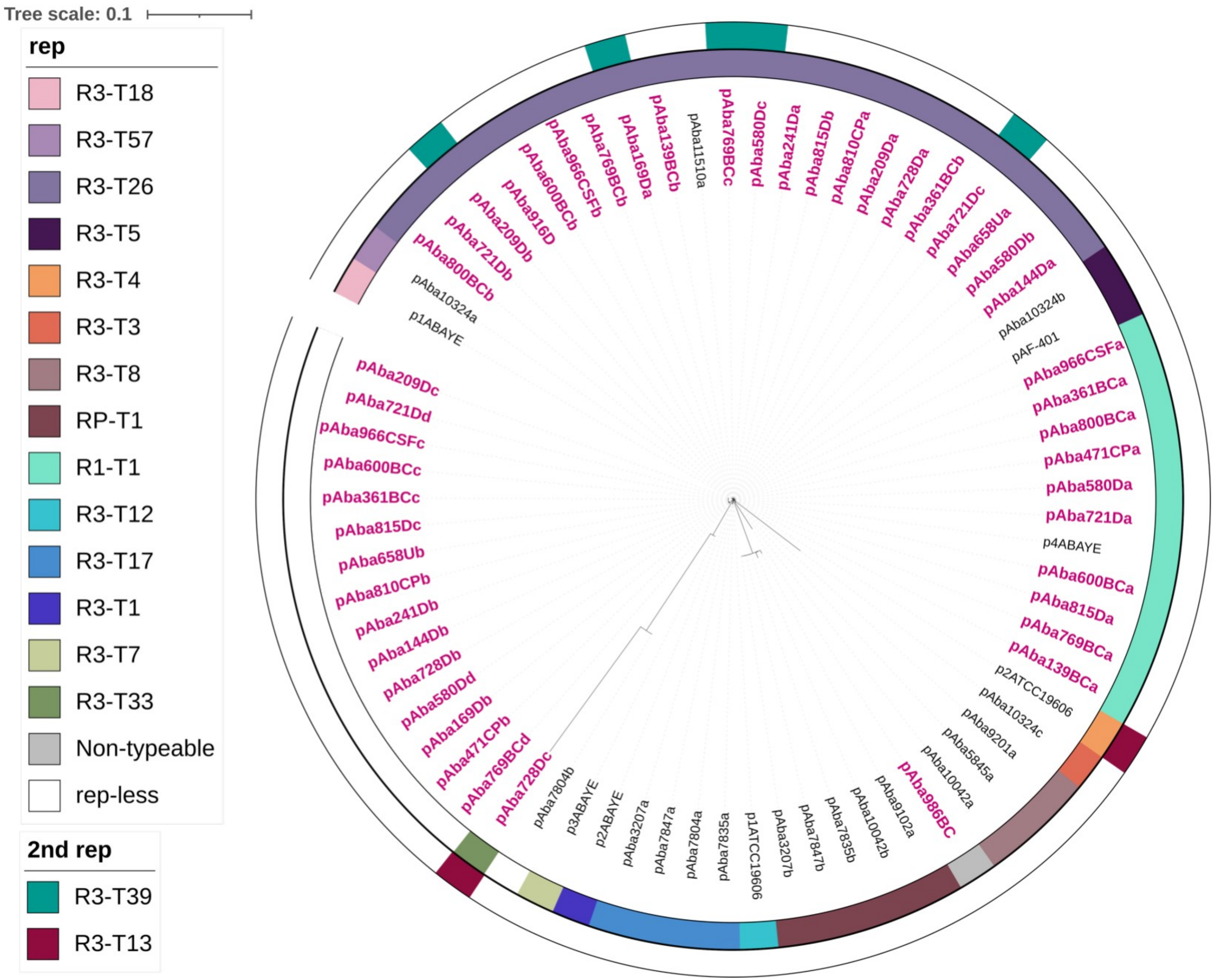

FIG S4 Pangenomic ML phylogeny for CRAB-HIMFG and AB-DB plasmids using *rep* gene based typing. CRAB-HIMFG plasmids are highlighted in bold pink text.
